# Supplementary material for: splatPop: simulating population scale single-cell RNA sequencing data
Source: Genome Biol. 2021 Dec 15;22:341. doi: 10.1186/s13059-021-02546-1 (PMC8672480; doi:10.1186/s13059-021-02546-1)
Supplement: Supplementary file 1 — Additional file 1 Supplementary Table S1-S2 and Supplementary Fig S1-S8 including legends. [file 13059_2021_2546_MOESM1_ESM.pdf]

Supplementary Materials

splatPop: simulating population scale single-cell RNA sequencing data

Azodi et al

Table of contents

Contents

Table of contents 2

Supplementary Tables 3

Supplementary Figures 6

Supplementary Tables

Table S1. Input parameters for the splatPop simulation model

ID	Estimated	Category	Symbol	Description	Default Value
			**Population parameters**		
pop.mean.shape	yes	population	α_m	Gamma shape for population-wide gene means	0.34
pop.mean.rate	yes	population	β_m	Gamma rate for population-wide gene means	0.008
pop.cv.param	yes	population	α_v, β_v	Data frame containing gamma shape and rate for population-wide gene variance by bin	varies
**eQTL parameters**					
eqtl.ES.shape	yes	eQTL	α_e	eQTL & Gamma shape for eQTL effect sizes	3.6
eqtl.ES.rate	yes	eQTL	β_e	eQTL & Gamma rate for eQTL effect sizes.	12
**Single-cell parameters** (estimated with original splatEstimate function)					
mean.shape	yes	single-cell	α_sc	Shape parameter for the mean gene expression gamma distribution	0.6
mean.rate	yes	single-cell	β_sc	Rate parameter for the mean gene expression gamma distribution	0.3
lib.loc	yes	single-cell	μ_L	Location parameter for the library size log-normal distribution	11
lib.scale	yes	single-cell	σ_L	Scale parameter for the library size log-normal distribution	0.2
out.prob	yes	single-cell	π_O	Probability that a gene is an expression outlier	0.05
out.facLoc	yes	single-cell	μ_O	Location parameter for the expression outlier factor log-normal distribution	4
out.facScale	yes	single-cell	σ_O	Scale parameter for the expression outlier factor log-normal distribution	0.5
bcv.common	yes	single-cell	φ	Common BCV dispersion across all genes in single-cell data	0.1
bcv.df	yes	single-cell	df_0	Degrees of freedom for the BCV inverse chi-squared distribution	60
dropout.mid	yes	single-cell	x_0	Midpoint for the dropout logistic function	0

dropout.shape	yes	single-cell	k	Shape of the dropout logistic function	-1
**Manual parameters**					
similarity.scale	no	population	s_s	Scaling factor for pop.cv.param.rate, where values larger than 1 increase the similarity between individuals in the population and values less than one make the individuals less similar.	1
pop.cv.bins	no	population		Number of gene mean bins to use to estimate CV params	10
pop.quant.norm	no	population		T/F if simulated gene means per individual should be quantile normalized to fit the distribution of the single-cell gene mean distribution	TRUE
eqtl.n	no	eQTL		Number (if >1) or proportion of genes to simulate as eGenes	1
eqtl.dist	no	eQTL		Maximum distance from center of eGene to eSNP	1 Mb
eqtl.maf.min	no	eQTL		Minimum minor allele frequency of eSNP	0.05
eqtl.maf.max	no	eQTL		Maximum minor allele frequency of eSNP	0.5
eqt.coreg	no	eQTL		Proportion of eGenes to have a shared eSNP (i.e. co-regulation)	0
eqtl.group.specific	no	eQTL		Proportion of eQTL to set as group specific if nGroups >1	0.2
eqtl.condition.specific	no	eQTL		Proportion of eQTL to set as condition specific if nConditions >1	0.2
nCells.sample	no	single-cell		T/F if nCells should be sampled from a gamma distribution for each batch/donor.	FALSE
nCells.shape	no	single-cell		Shape parameter for the nCells per batch per donor distribution.	1.5
nCells.rate	no	single-cell		Rate parameter for the nCells per batch per donor distribution.	0.015
batch.size	no	batch effects		The number of donors in each pool/batch.	10
batchCells	no	batch effects		A vector specifying the number of cells per individual in each batch. This is ignored if nCells.sample=TRUE.	
batch.facLoc	no	batch effects	μ_b	Location (meanlog) parameter for the batch factor log-normal distribution.	0.1
batch.facScale	no	batch effects	σ_b	Scale (sdlog) parameter for the batch factor log-normal distribution.	0.1

de.prob	no	group effects	π_de	Probability that a gene is DE in a cell group.	0.1
de.downProb	no	group effects		Probability that a group-DE gene is down-regulated.	0.5
de.facLoc	no	group effects	μ_de	Location (meanlog) parameter for the group-DE factor log-normal distribution.	0.1
de.facScale	no	group effects	σ_de	Scale (sdlog) parameter for the group-DE factor log-normal distribution.	0.4
nConditions	no	conditional effects		The number of conditions/treatments to divide samples into.	1
condition.prob	no	conditional effects		Probability that a sample belongs to each condition/treatment group.	c(0.5, 0.5)
cde.prob	no	conditional effects	π_cde	Probability that a gene is DE in a conditional cohort.	0.1
cde.downProb	no	conditional effects		Probability that a conditional-DE gene is down-regulated.	0.5

Table S2. Time (minutes) to simulate populations with N individuals (rows) and N genes (columns), with 100 cells from a single cell-group simulated per individual.

		**# genes**					
		**10**	**100**	**500**	**1000**	**10k**	**20k**
**# individuals**	**10**	0.05	0.09	0.32	0.60	5.80	11.71
	**100**	0.30	0.34	0.56	0.84	6.08	12.12
	**100***	0.33	0.34	0.57	0.82	5.92	11.99
	**500**	1.43	1.48	1.75	2.09	8.67	16.23

* simulated in 10 batches with 10 individuals per batch.

Supplementary Figures

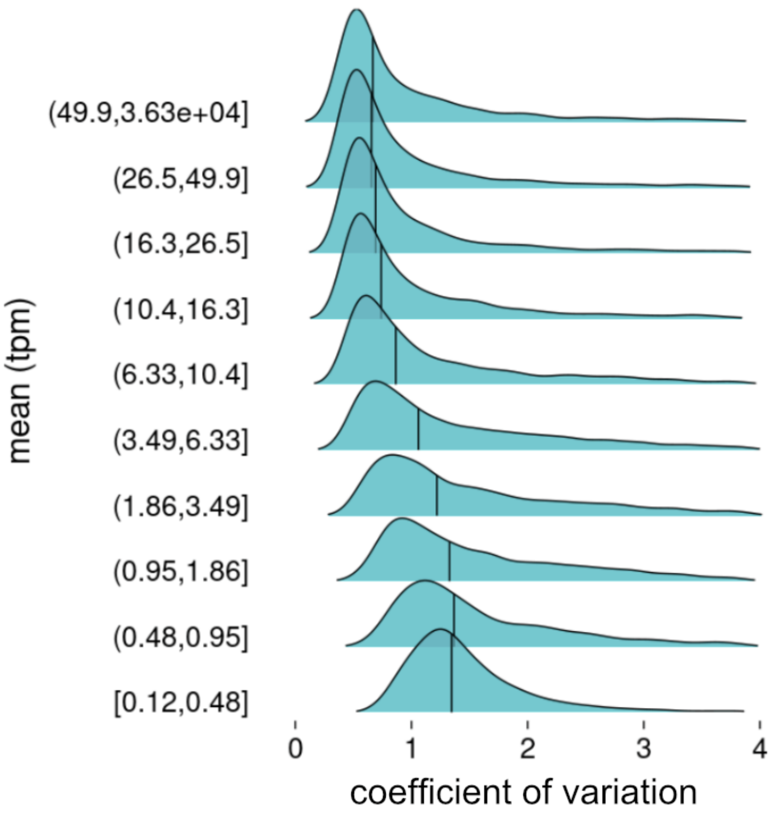

Fig. S1. The gene mean-variance trend. The distribution of gene expression coefficient of variation between individuals, binned by gene mean across individuals (y-axis). Expression data from GTEx thyroid tissue. The bar indicates the median variance per expression bin. tpm: transcripts per million.

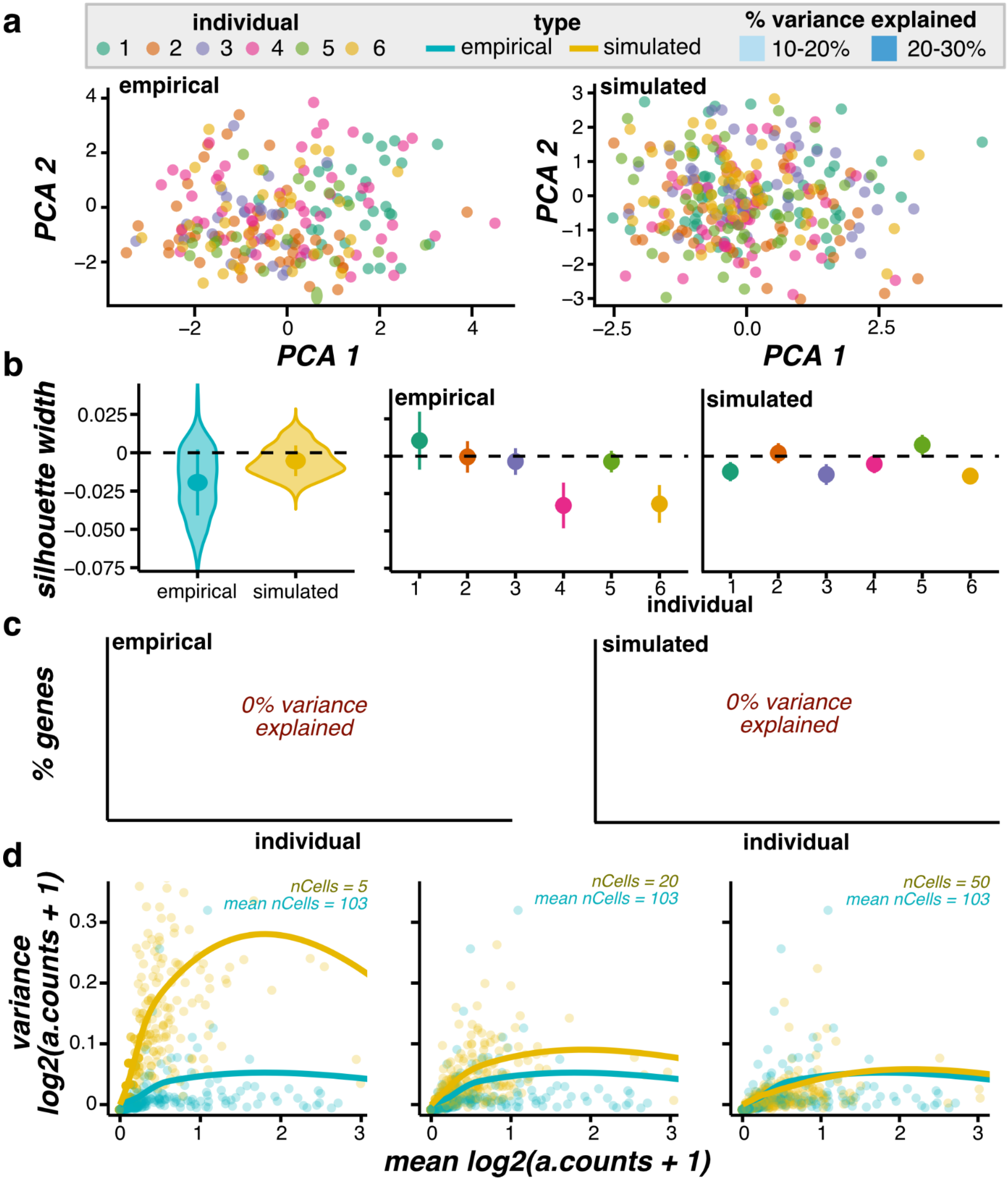

Fig. S2. Simulated compared to empirical 10x-Neuro single-cell RNA-seq data. (a) PCA plots of cells colored by individual. **(b)** The distribution of cell silhouette widths using the individual as the cluster. The distributions are shown for cells grouped by type (left) and by type and individual (right), with the point and whisker showing the mean and standard deviation. **(c)** The percent of genes (y-axis) with a given percentage of variance explained by individual. Note, no variance in gene expression was explained by individual in the 10x-Neuro empirical or simulated data. **(d)** The mean-variance relationship across a range of nCells simulated per individual. The counts per gene were mean-aggregated (a.counts) across nCells from each individual and logged before calculating the population wide mean and variance. All cells were used from the empirical data (average = 103), while simulated data was down-sampled to nCells per individual.

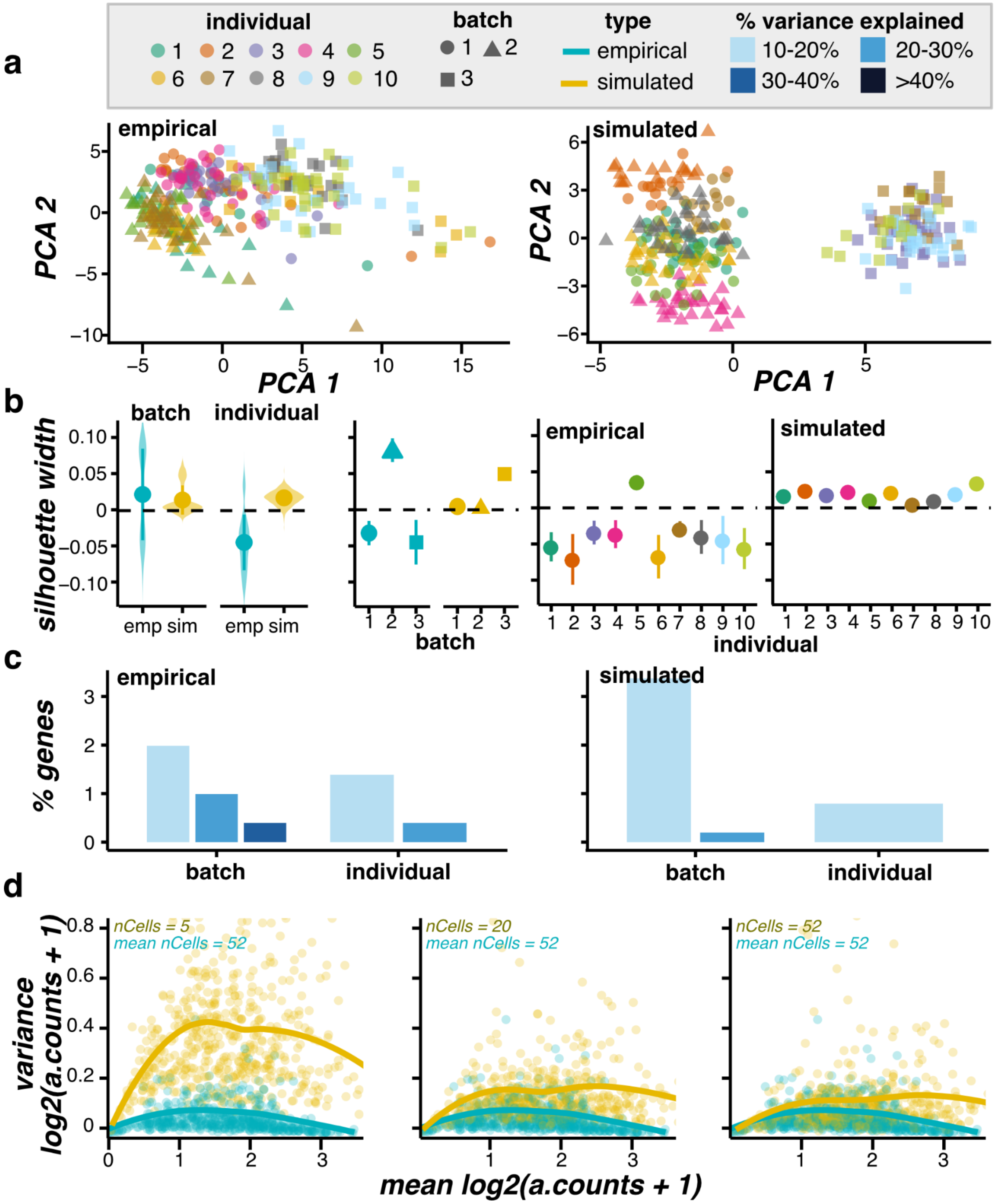

Fig. S3. Simulated compared to empirical SmartSeq2 iPSC single-cell RNA-seq data (ss2-iPSC) from three batches. (a) PCA plots of cells colored by individual and shaped by batch (max 50 cells shown per individual). **(b)** The distribution of cell silhouette widths using the batch or individual as the cluster. The distributions are shown for cells grouped by type (left) and by type, batch (middle), and individual (right), with the point and whisker showing the mean and standard deviation. **(c)** The percent of genes (y-axis) with a given percentage of variance explained by batch and individual. **(d)** The mean-variance relationship across a range of nCells simulated per individual. The counts per gene were mean-aggregated (a.counts) across nCells from each individual and logged before calculating the population wide mean and variance. All cells were used from the empirical data (average = 52), while simulated data was down-sampled to nCells per individual.

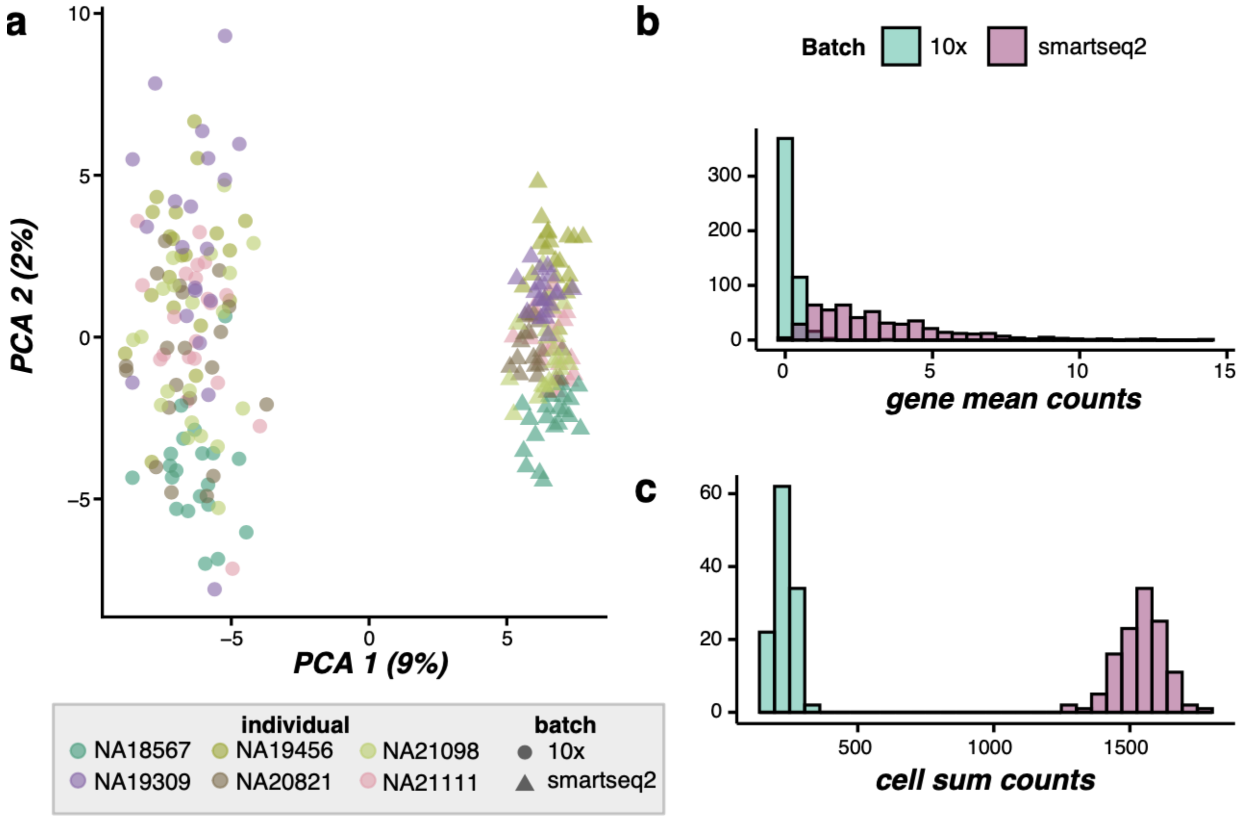

Fig. S4. Simulated cells from different chemistries. (a) PCA plots of cells colored by individual and shaped by chemistry batch (n=20 cells per individual per batch). The distribution of **(b)** gene mean counts and **(c)** cell count sums between the two chemistries.

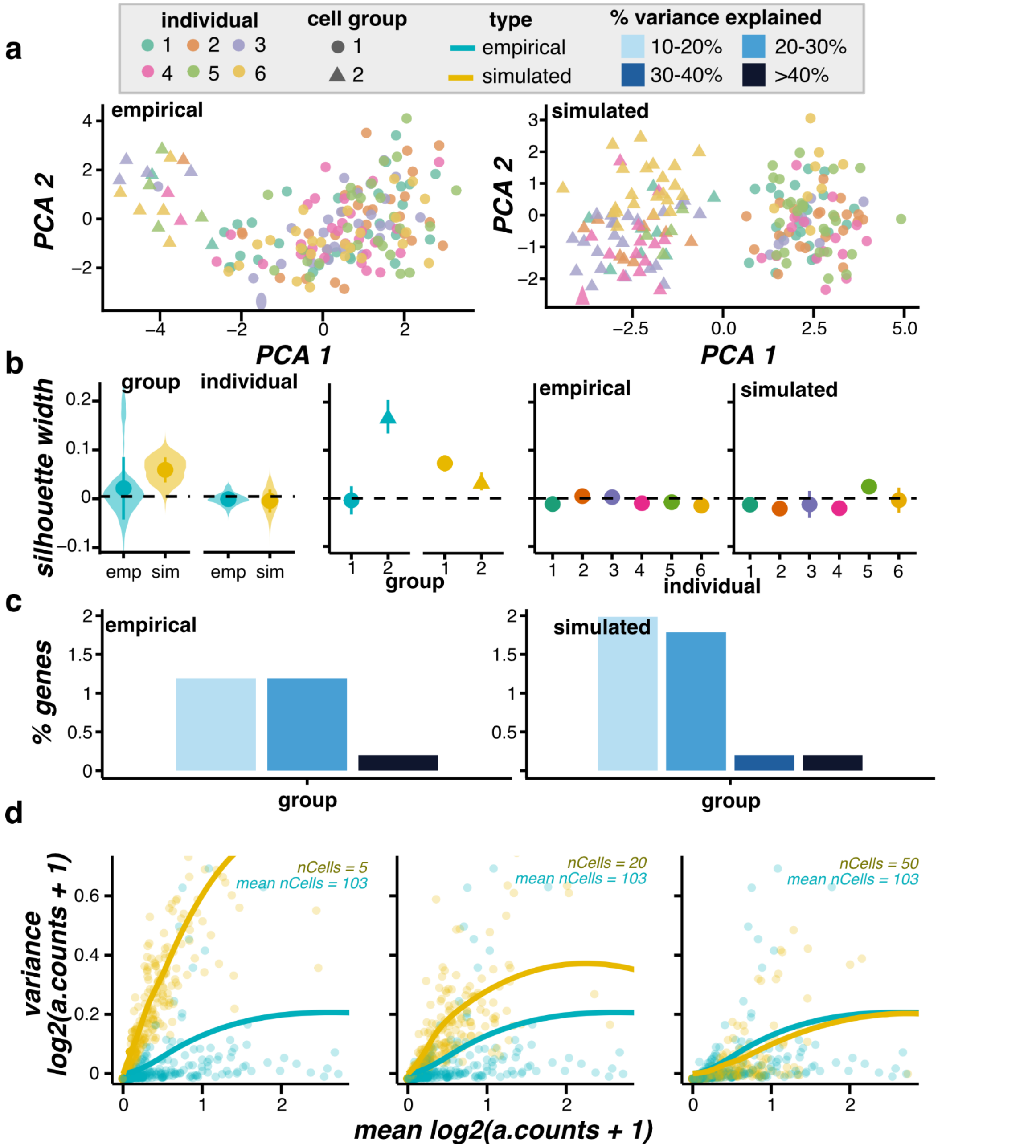

Fig. S5. Simulated compared to empirical 10x-Neuro data from floor plate progenitor and dopaminergic neuron cells. (a) PCA plots of cells colored by individual and shaped by cell group (max 50 cells shown per individual). **(b)** The distribution of cell silhouette widths using the cell group or individual as the cluster. The distributions are shown for cells grouped by type (left) and by type, cell group (middle), and individual (right), with the point and whisker showing the mean and standard deviation. **(c)** The percent of genes (y-axis) with a given percentage of variance explained by cell group and individual. Note, no variance in gene expression was explained by individual in the 10x-Neuro empirical or simulated data. **(d)** The mean-variance relationship across a range of nCells simulated per individual. The counts per gene were mean-aggregated (a.counts) across nCells from each individual and logged before calculating the population wide mean and variance. All cells were used from the empirical data (average = 103), while simulated data was down-sampled to nCells per individual.

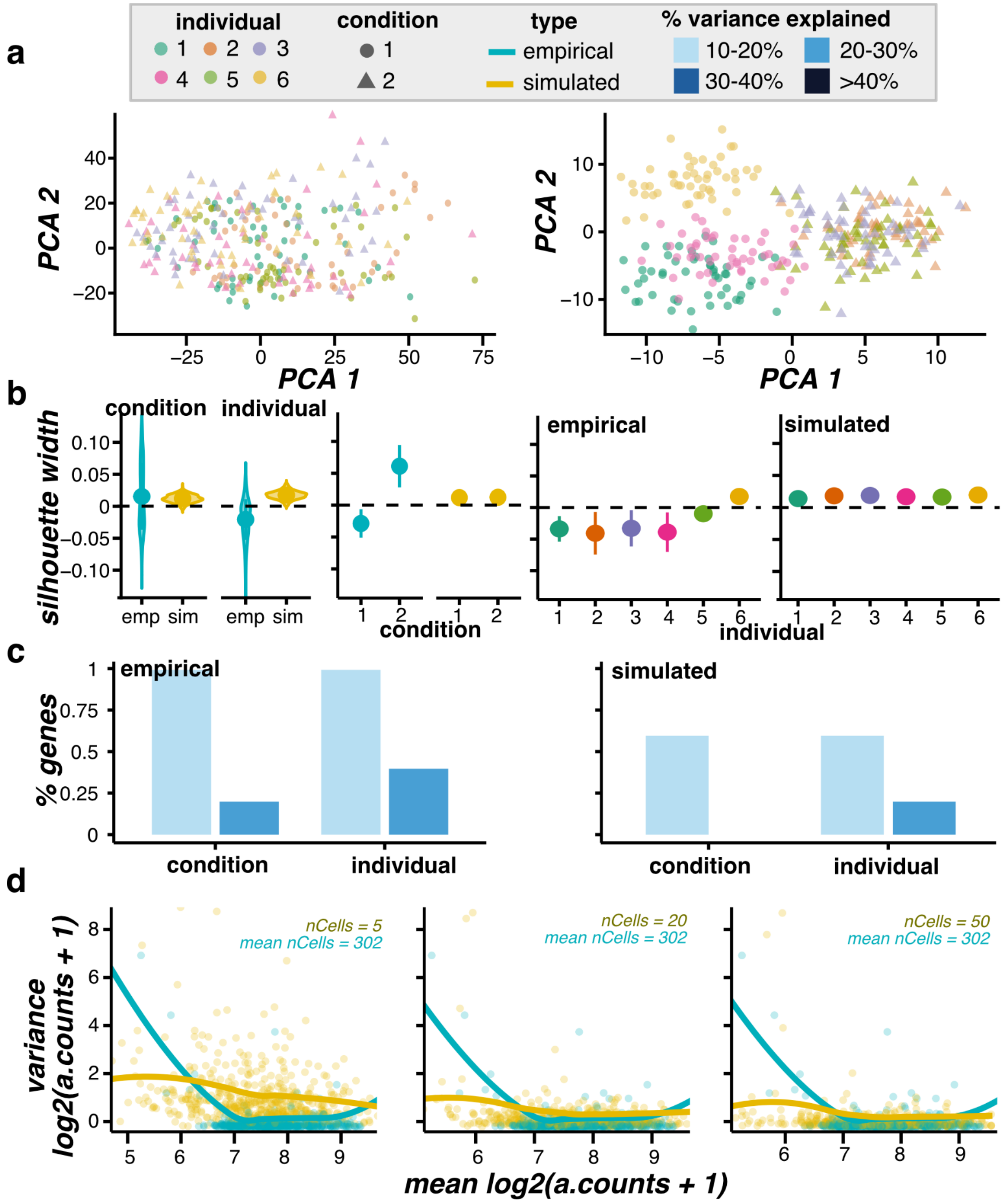

Fig. S6. Simulated compared to empirical 10x-IPF single-cell RNA-seq data from fibroblast cells for 3 healthy and 3 stimulated IPF samples. (a) PCA plots of cells colored by individual and shaped by conditional group. **(b)** The distribution of cell silhouette widths using the conditional group or individual as the cluster. The distributions are shown for cells grouped by type (left) and by type, conditional group (middle), and individual (right), with the point and whisker showing the mean and standard deviation. **(c)** The percent of genes (y-axis) with a given percentage of variance explained by conditional group and individual. **(d)** The mean-variance relationship across a range of nCells simulated per individual. The counts per gene were mean-aggregated (a.counts) across nCells from each individual and logged before calculating the population wide mean and variance. All cells were used from the empirical data (average = 302), while simulated data was down-sampled to nCells per individual.

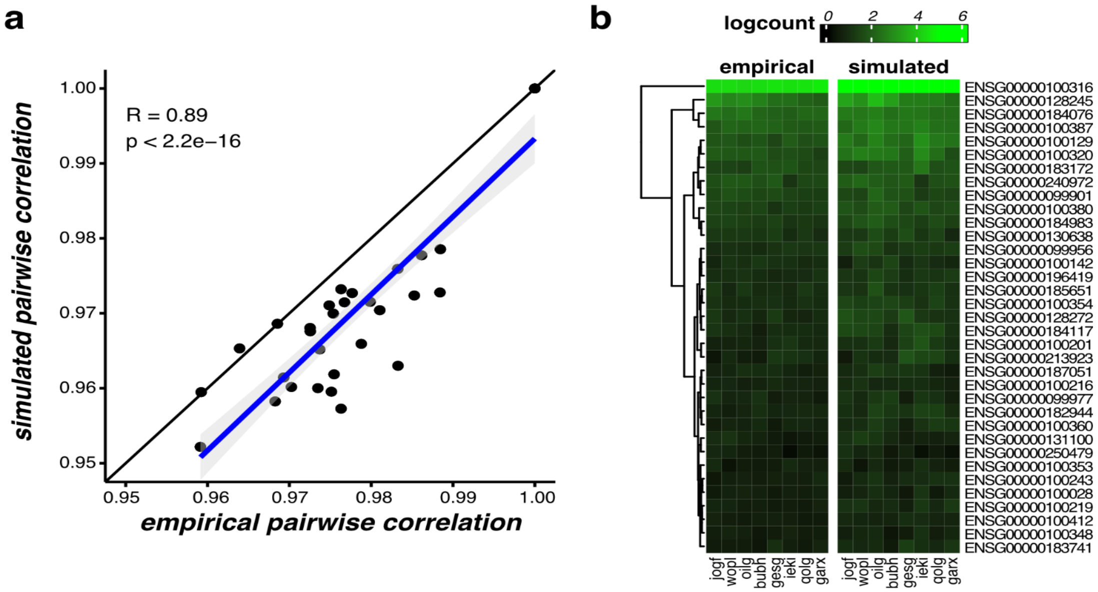

Fig. S7. Additional comparisons of 10x-Neuro and splatPop simulated data using the replication approach. (a) The relationship between the pairwise Pearson's correlation between individuals across genes (expression for each gene for each individual calculated as the mean aggregated log counts across cells) from empirical (x-axis) and simulated (y-axis) data. The diagonal is shown with the black line, the linear regression line is shown with the dashed blue line is the diagonal, the Pearson's correlation between empirical and simulated pairwise correlation is shown in the top left corner. **(b)** Mean aggregated log counts across cells for each individual for the top 35 most highly expressed genes. Genes and individuals are ordered by hierarchical clustering of the empirical data.

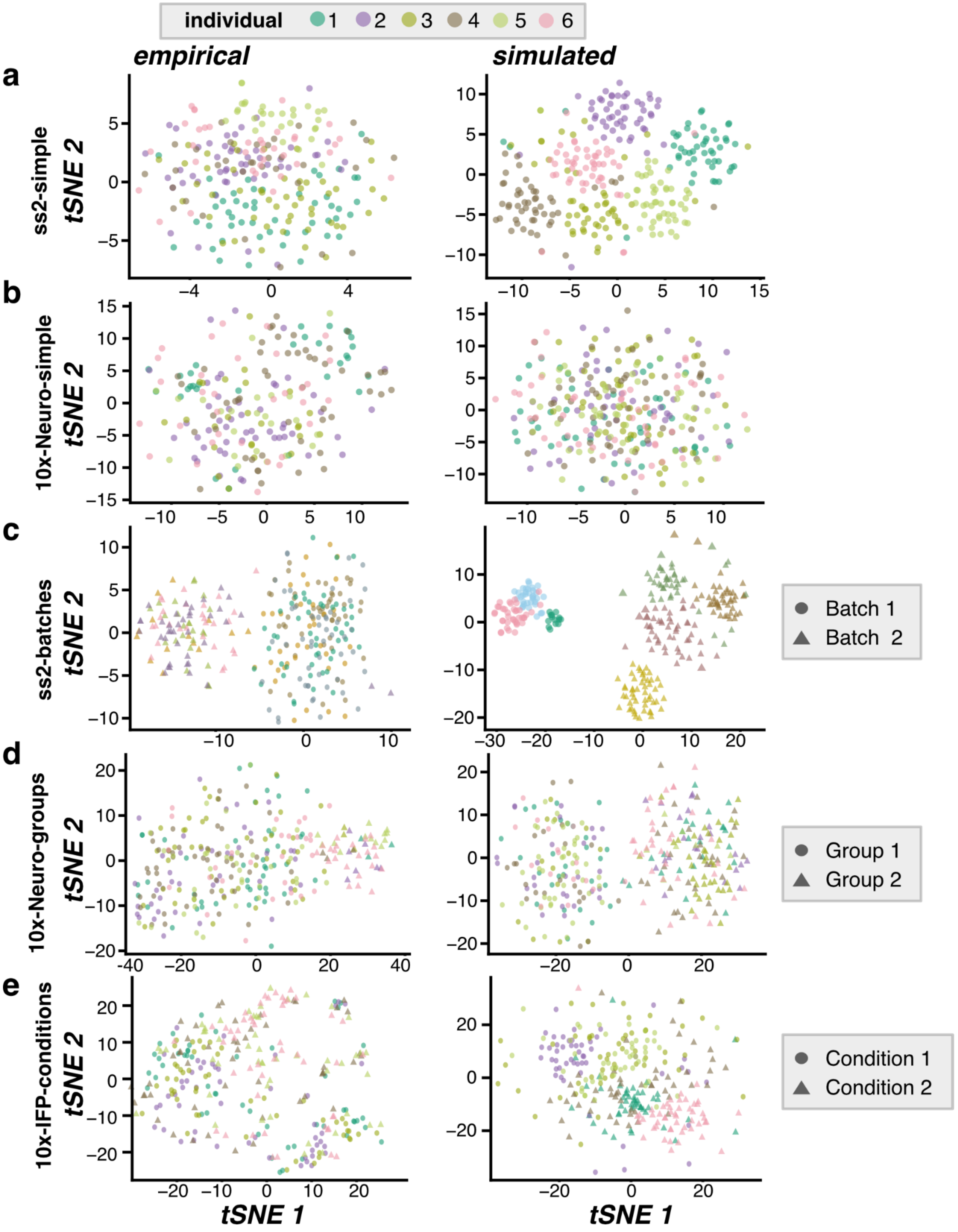

Fig. S8. Visualizing simulated compared to empirical single-cell RNA-seq data using tSNE plots. tSNE dimension reduction was performed using PCA results as input with functions from scater (seed=42, perplexity=20) on empirical (left) and simulated (right) data from **(a)** SmartSeq2-iPSCs, **(b)** 10x-Neuro cells, **(c)** SmartSeq2-iPSCs with batch effects, **(d)** 10x-Neuro cells with group effects, and **(e)** 10x-IPF cells with conditional effects.

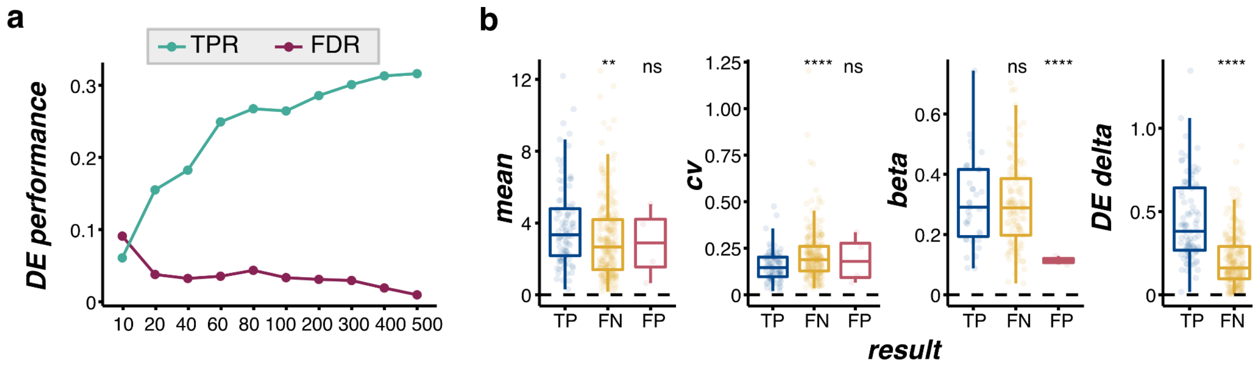

Fig. S9. Wilcoxon rank sum DE test results. Pseudo-bulk differential expression (DE) analysis using the Wilcoxon rank sum test with BH false discovery rate correction. (**a**) The true positive rate (TPR: TP/(TP+FN)) and false discovery rate (FDR: FP/(TP+FP)) of DE genes (q.value < 0.05) between two conditional groups across a range of number of simulated cells per individual (x-axis) using 10x-IPF as a reference. (**b**) The simulated gene mean and coefficient of variation (cv), eQTL effect size (beta, if applicable to that gene), and DE effect size (DE delta) for TP, FN, and FP DE genes using 80 cells per individual. Statistical significance is reported for t-tests testing for difference between TP and FN or FP categories (ns: p > 0.05, *: p <= 0.05, **: p <=, 0.01, ***: p <= 0.001, ****: p <= 0.0001).
