# Supplementary material for: splatPop: simulating population scale single-cell RNA sequencing data
Source: Genome Biol. 2021 Dec 15;22:341. doi: 10.1186/s13059-021-02546-1 (PMC8672480; doi:10.1186/s13059-021-02546-1)
Supplement: Supplementary file 2 — Additional file 2 Review history. [file 13059_2021_2546_MOESM2_ESM.docx]

Review History

**First round of review**

**Reviewer 1**

**Were you able to assess all statistics in the manuscript, including the appropriateness of statistical tests used?**

Yes: Statistics are fine, the only thing that deviates from the standard: - Page 12, line 59: why is a significance cutoff of FDR of 0.10 used, and not the more common significance 0.05 cutoff?

**Were you able to directly test the methods?**

No

**Comments to author:**

The authors present a framework to simulate genetic and complex batch effects for modelling population-scale scRNA-seq data. Such a tool is currently lacking and may be used in the future to test newly developed scRNA-seq tools or benchmark tools against each other. Certain aspects however, need further experiments to show the broader applicability and correct simulation of for example co-expression relationships.

Major points:

- Page 5, line 56: why did you not test if supplying the genotype data, actually gave you the expected interindividual relationships? Please provide an additional graph to Fig. 2B in which the simulated dataset contains the same genotype info as the empirical dataset.

- Page 6, line 8: please show the ncells per donor vs the mean-variance relationship in figure 2 and figure 3, as this will help define the number of cells required to obtain stable mean-variance relationships. And so to know whether the presented results have sufficient cells per donor to be confident in what is presented.

- Figure 2: It is unclear what the added benefit is of the 'simple' model, the 'complex' model seems to always work better in real-life settings. Would you not always want have the best representation of an actual dataset? Perhaps focus on the 'complex' model, and move the 'simple' model to the supplements.

- Instead of presenting the 'simple' model in the main text, the paper would greatly be improved by expanding the 'complex' model part. Something that has not been mentioned at all, but is very relevant is the correct modelling of co-expression relationships, and coexpression-QTLs (i.e. co-expression relationships affected by genetics). Please expand the 'complex' model part with a section on how well the model correctly preserves gene-gene relationships and include tests with gene network reconstruction tools.

- Page 7, line 5: Some batch effects, such as when combining datasets of different 10x chemistries, show non-linear relationships. Can you expand the model in such a way that these batch effects can also be modelled? This would further improve the applicability of the tool.

- Page 8, line 29: Your tool models scRNA-seq data, and therefore, to define how well the single-cell data is modelled it makes more sense to also show a single-cell specific DE method that actually takes into account the properties of the single-cell data. So in addition to the Wilcoxon rank sums test, I would like to see the performance with a commonly used single-cell specific DE tool such as MAST.

- Page 7, line 10: The number of individuals being multiplexed in a 10x experiment seems to be increasing especially in the context of population-scale scRNA-seq data (e.g. through new developments like https://www.nature.com/articles/s41592-021-01153-z?proof=t%29). Therefore, it would be relevant to the reader to know how the performance is affected with different numbers of samples (10s instead of 5). Please show how the tool performs/compare multiplexing of 10s of individuals with the 5 shown now.

- Page 11, line 20: why specifically this cutoff? Please show benchmarks with commonly used parameters (such as 'expressed in at least 10% of cells in one condition' in MAST, or using all genes that are expressed and show variance in eQTL mapping).

Minor points:

- Page 2, line 36: Perhaps the wording 'top-tier' is a bit overselling the tool. While it is ranked high in the referenced paper, it is not without its downsides and is definitely not top-tier for keeping 'biological signals'.

- Figure 1: the parameters 'BatchCells' and 'Batch.size' are missing here. Table S1 is also missing 'BatchCells'. Please show how these parameters fit into the model and add 'BatchCells' parameter to Table S1.

- Page 4, line 18: write out the abbreviation 'BCV' completely the first time it's mentioned.

- Page 7, line 31: spelling error 'versus', not 'verses'

- Page 12, line 59: why is a significance cutoff of FDR of 0.10 used, and not the more common significance 0.05 cutoff?

**Reviewer 2**

**Were you able to assess all statistics in the manuscript, including the appropriateness of statistical tests used?**

Yes: As a statistician, I am able to comment on the methodology and statistics used in this manuscript.

**Were you able to directly test the methods?**

Yes

**Comments to author:**

In this study, the authors proposed a novel simulation method that generate scRNA-seq data with corresponding eQTL effect. The same simulation framework also allows for the simulation of complex batch effects, cell group effects, and conditional effects between individuals from different cohorts. The manuscript is well-written and the models are presented very clearly with visually pleasing figures and reproducible html. While the concept simulating eQTL effect in the model is innovative, we feel the current version of the manuscript as it stands didn't fully validate the simulation model. The manuscript will benefit from further clarity and justification of the model. Below we comment and suggest a number of steps that will add to the manuscript.

1. There are two key components or application aspects of the paper (a) Creating a new simulation model that can simulate population-scale scRNA-seq data with corresponding eQTL effect. (b) The additional ability of this model to generate scRNA-seq data with more complex intrinsic structure. It is important to validate the two aims of this paper separately.

2. Following on from point 1 (specially component 1b), its important to compare more widely. A recent simulation framework, Muscat*, also enables the simulation of multi-samples across multi-conditions. While this method has a different framework and does not use eQTL data in its modelling, the authors may want to consider citing and discussing this work. * Crowell, H. L., Soneson, C., Germain, P. L., Calini, D., Collin, L., Raposo, C., ... & Robinson, M. D. (2020). Muscat detects subpopulation-specific state transitions from multi-sample multi-condition single-cell transcriptomics data. Nature communications, 11(1), 1-12.

3. In Fig 2a, the authors showed that the inter-individual relationship pattern between empirical and simulated individuals is not the same, and stated it is because the simulated individuals do not have the same genotype as the reference individual. The authors then mentioned, "However, such relationship patterns could be achieved by providing splatPop with the genotype data for the population of interest." As the novelty of splatPop lies in its ability to take in genotype data and simulate realistic population structure, an example case study of the above statement should be provided. Currently, it seems that in majority of case studies (Fig 2, Fig S2-S5), the inter-individual relationship pattern in the simulated data is different to the empirical.

4. The authors referred to a recent benchmark ms on scRNA-seq simulation methods*. From the paper, while Splatter is one of the top-tier methods in general across 13 criteria under data property estimation, it performed medicorely in certain criteria such as cell correlation, mean vs fraction of zero relationship and library size vs fraction zero relationship (Fig 2.). While this seems to be a challenging criteria for many simulation methods in general was splatPop able to address this issue and improve from Splat on some of these criteria? * Cao, Y., Yang, P., & Yang, J. Y. H. (2021). A benchmark study of simulation methods for single-cell RNA sequencing data. bioRxiv.

5. Relating to the previous comment, the benchmark paper utilised a strategy of splitting an empirical data into training and testing subset, using the training to simulate and the testing to evaluate the simulated data. A similar framework could be useful to evaluate the simulated data generated in this study and to complement the visuals. For example, given that the data used for simulation in this study includes eQTL information, are the authors able to compare the eQTL effec ts generated in the simulated data with the empirical eQTL effects?

6. Following on point 5, the authors provided a case study of how splatPop can utilise genetic effects on expression (eQTL) to define cell groups by genetic and non-genetic DE. It would be good to extend this case study by providing a downstream of application of this simulated data. Ie, what can people do with the data besides performing a typical DE analysis and finding DE genes between two groups? This allow the comparison of another downstream performance between empirical and simulated data.

7. When using splatPop to evaluate DE and eQTL methods, the authors set the conditional DE effects on 40% of the genes and eQTL effect to 70% of genes. These numbers seem quite high, is there a particular reason for choosing such high percentages? Would setting it to a lower number, say 10%, impact the DE and eQTL results?

8. Given that a scRNA-seq typically contains more than 20,000 genes, and that a user may want to simulate genes from all chromosomes rather than from just one chromosome, the authors may want to expand their scalability analysis in Table S2 to include more genes. At the moment, only up to 1000 genes is examined.

9. (minor) In Table S2, the table legend indicates the time is in minutes. However, from reading the manuscript it should be in seconds?

10. The benefit of using eQTL data as an extra source of information to simulate multiple individuals needs to be emphasised in greater detail. May be further discussion and additional case studies.

**Authors Response**

**Point-by-point responses to the reviewers’ comments:**

Reviewer #1:

The authors present a framework to simulate genetic and complex batch effects for modelling population-scale scRNA-seq data. Such a tool is currently lacking and may be used in the future to test newly developed scRNA-seq tools or benchmark tools against each other. Certain aspects however, need further experiments to show the broader applicability and correct simulation of for example co-expression relationships.

*We thank the reviewer for their time in assessing our manuscript and for their thoughtful comments, addressed below.*

Major points:

1. Page 5, line 56: why did you not test if supplying the genotype data, actually gave you the expected interindividual relationships? Please provide an additional graph to Fig. 2B in which the simulated dataset contains the same genotype info as the empirical dataset.

*Due to the sampling performed by splatPop (i.e. when gene means for each individual are sampled from a gamma distribution parameterized from empirical data), splatPop simulations maintain the population-level properties of empirical data, but not the inter-individual relationships. While this approach has advantages, like being fast and being able to simulate larger datasets than what you have available empirically, this comment from the reviewer (and comment #3 from reviewer #2) encouraged us to extend the functionality of splatPop to allow users to more closely replicate provided empirical data if desired. This new function works by having users provide the empirical data directly to the splatPopSimulate function (before it was provided only to the splatPopEstimate function), thereby skipping the gene mean and variance sampling steps, and using the provided data directly as the base gene means. An example of how to use this feature has been added to the package vignette and the provided workflowr scripts that support this manuscript. In addition, the following new results section and supporting figure have been added to the manuscript:*

*Replicating empirical data with splatPop: Without the addition of co-regulation effects, the expression correlation between genes is centered around zero (Figure 4a). This effect occurs because the mean expression value for each gene for each individual is sampled randomly from a distribution. This sampling approach is fast, flexible (e.g., allowing for the simulation of any number of genes), and it maintains the population-level characteristics of empirical scRNA-seq data, all useful properties for many simulation studies. However, splatPop can also be used to simulate scRNA-seq data that more closely replicates the gene expression correlations seen in empirical data. As described above, splatPop estimates population-wide parameters from user provided population-scale bulk RNA-seq or aggregated scRNA-seq data. However, if this empirical data is provided directly to the splatPopSimulate function, it will be used as the base gene means (i.e. the "Gene mean per sample" Figure 1, left panel). Used in this way, splatPop will generate simulated scRNA-seq data for the individuals and genes in the empirical data that replicates empirical inter-individual relationships (Figure 4b) and patterns of gene expression across individuals (Figure 4c). We note that while cell-group, conditional, and batch effects can still be applied to simulations generated this way, adding simulated eQTL effects may compound with eQTL effects already present in the empirical gene mean data, inflating their effect size.*

*Methods: We note that if empirical gene mean data is provided directly to the splatPopSimulate function, the gene mean and variance sampling steps will be ignored, and the empirical gene means will be used directly.*

*Figure 4 Co-regulation and replicating empirical 10x-Neuro data. (a) Pearson's correlation between mean aggregated expression of randomly paired (gray, n=1000) and co-regulated (blue, n=54) eGenes across 100 individuals. The average correlation for each type designated by the large point. (b, c) Comparison of 10x-Neuro (left) and splatPop simulated (right) data using the replication approach. Eight individuals in the same batch and 358 genes meeting a minimum expression threshold (> 10 counts across all cells) were included. (b) Pairwise Pearson's correlation between individuals across genes, with expression for each gene for each individual calculated as the mean aggregated log counts across cells. Individuals are ordered by hierarchical clustering of the empirical data. (c) Mean aggregated log counts across cells for each individual for the top 35 most highly expressed genes. Genes and individuals are ordered by hierarchical clustering of the empirical data.*

2. Page 6, line 8: please show the ncells per donor vs the mean-variance relationship in figure 2 and figure 3, as this will help define the number of cells required to obtain stable mean-variance relationships. And so to know whether the presented results have sufficient cells per donor to be confident in what is presented.

*The number of cells simulated per donor varies by simulation. Taking the suggestion from the reviewer, we have updated panel (d) in all simulation vs. empirical comparison Figures (Figures 2, S2, S3, S5, and S6) to demonstrate that the mean-variance relationship stabilizes when more cells are simulated. We also note that we also updated the metric used to show variance (originally coefficient of variation, now variance) to match what is used in the original splatter manuscript and in Cao et al. This does not change the interpretation, but shows the relationship in a way we believe is more familiar.*

*Here we compare the mean-variance relationship for each gene across individuals, with the counts per gene per individual calculated by mean aggregating counts across cells (see Methods; Figure 2d). From left to right, the plots show that the mean variance relationship stabilizes as more cells are simulated per individual. We also demonstrate that splatPop can simulate a simple data set with the cell-level and gene-level properties of data generated on the 10x sequencing platform (Figure S2). This example better demonstrates the stabilization of the mean variance trend as the number of simulated cells increases (Figure S2d).*

*Figure 2d:*

*Figure 2 [...] (d) The mean-variance relationship. The counts per gene were mean-aggregated (a.counts) across nCells from each individual and logged before calculating the population wide mean and variance. All cells were used from the empirical data (average = 52), while simulated data was down-sampled to nCells per individual.*

*Figure S2d:*

*Figure 2 [...] (d) The mean-variance relationship across a range of nCells simulated per individual. The counts per gene were mean-aggregated (a.counts) across nCells from each individual and logged before calculating the population wide mean and variance. All cells were used from the empirical data (average = 103), while simulated data was down-sampled to nCells per individual.*

3.	Figure 2: It is unclear what the added benefit is of the 'simple' model, the 'complex' model seems to always work better in real-life settings. Would you not always want have the best representation of an actual dataset? Perhaps focus on the 'complex' model, and move the 'simple' model to the supplements.

*We agree that, in most cases, splatPop users will want to simulate complex single-cell RNA-seq populations. However, the benefit of starting the paper with a simple simulation is that it allows us to introduce the basic functionality of splatPop and demonstrate how we compare simulations to empirical data before getting into all of the advanced features. We worry that jumping straight into complex simulations without establishing the basic principles will make the manuscript difficult to read. We would also note that the simple simulation represents actual data as well as the complex simulations, it just represents data generated with a less complex experimental design. For example, the simple simulation in Figure 2 could represent real scRNA-seq data from a homogenous cell population (e.g. iPSCs) from six healthy individuals all sequenced in one batch; such experiments are not uncommon. We have kept the simple simulation as our first example, but if the editor agrees that the simple simulation is not necessary for describing the tool we are happy to move it to the supplement.*

4.	Instead of presenting the 'simple' model in the main text, the paper would greatly be improved by expanding the 'complex' model part. Something that has not been mentioned at all, but is very relevant is the correct modelling of co-expression relationships, and coexpression-QTLs (i.e. co-expression relationships affected by genetics). Please expand the 'complex' model part with a section on how well the model correctly preserves gene-gene relationships and include tests with gene network reconstruction tools.

*We appreciate the suggestion to highlight co-regulation relationships between genes in our manuscript. By default, splatPop randomly assigns eSNPs to eGenes (within the specified window size). However users can control what eQTL are simulated by either providing splatPop with (1, an existing feature) a splatPop key that specifies what eQTL associations should be simulated or (2, a new feature) empirical eQTL mapping summary statistics. If the splatPop key or the summary statistics provided have the same eSNP for multiple eGenes, the simulated data will have these genetic driven co-expression relationships. In response to the reviewer’s suggestion, we have also added a parameter to splatPopParams, called eqtl.coreg, that defines the percent of eGenes the user wants to be coregulated. For example, if eqtl.coreg is set to 0.1, splatPop will randomly select 10% of the eGenes, pair them with their nearest neighbor from that random set, and assign both in the pair the same eSNP. We ensure that eQTL that share an eSNP are given the same effect sign (i.e. all positive or all negative). We have added a section to the “Complex Simulations” results to describe all three of these approaches for generating genetic diven co-expression, demonstrate co-regulated genes in new Figure 4a, describe these functions in the Methods, and added this new parameter to Table S1:*

*Simulating genetically-driven co-expression: By default, splatPop randomly pairs eSNPs with eGenes (within the designated window), however splatPop can also be instructed to assign pairs of eGenes the same eSNP. This results in simulated scRNA-seq data with genetically-driven co-expression (i.e. co-regulation) relationships between genes. This can be done by providing eQTL data either as summary statistics or as a formatted splatPop key with the desired eQTL associations. Alternatively, the eqtl.coreg parameter can be set to the desired proportion of co-regulated eGenes. For example, in a simulation using the 10x-Neuro data as reference, setting eqt.coreg to 0.2 means that 20% of eGenes will be paired and assigned the same eSNP. These co-regulated eGenes have higher expression correlation across individuals than random pairs of eGenes (Figure 4a).*

Methods: Various control parameters can also be adjusted, including [...] and the percent of eGenes to have the same eSNP as another eGene (i.e., percent of genes with genetic co-regulation).

*Figure 4a. Gene-gene expression correlation. Pearson's correlation between mean aggregated expression of randomly paired (grey, n=1000) and co-regulated (blue, n=54) eGenes across 100 individuals. The average correlation for each type designated by the large point.*

5. Page 7, line 5: Some batch effects, such as when combining datasets of different 10x chemistries, show non-linear relationships. Can you expand the model in such a way that these batch effects can also be modelled? This would further improve the applicability of the tool.

*Having the batch effect function automatically simulate cells sequenced with different chemistries is not possible given that splatPop relies heavily on information provided in the splatPopParams object, which contains the parameters estimated from a homogenous population of cells (i.e., from a single chemistry). However, because splatPop is modular and reproducible, it is possible to generate cells with different properties using the splatPop key generated in another run, and merge these datasets together. We agree with the reviewer that this is an important application of splatPop, so we have added a code demonstration of how to do this to the workflowR document and have added the following to the results section on batch effects:*

*The modular and reproducible nature of splatPop simulations also allows for the simulation of multiple populations of cells that share "biological" signals (i.e., the same DE and eQTL effects) but differ in their single-cell properties. This feature can be useful to generate datasets made up of cells sequenced using different chemistries for different batches (Figure S4).*

*Figure S4. Simulated cells from different chemistries. (a) PCA plots of cells colored by individual and shaped by chemistry batch (n=20 cells per individual per batch). The distribution of (b) gene mean counts and (c) cell count sums between the two chemistries.*

6.	Page 8, line 29: Your tool models scRNA-seq data, and therefore, to define how well the single-cell data is modelled it makes more sense to also show a single-cell specific DE method that actually takes into account the properties of the single-cell data. So in addition to the Wilcoxon rank sums test, I would like to see the performance with a commonly used single-cell specific DE tool such as MAST.

*We have repeated our DE analysis using MAST as suggested by the reviewer. The general patterns observed (e.g. True positive DE genes increased with increased sample size and with increased DE delta), therefore we have moved the results from the Wilcoxon rank sum test to the supplement and put the results from MAST in Figure 4a, b in the main text.*

*snapshot of Figure 4a, b, c:*

*The following text was added to the methods to describe MAST: For a number of cells per individual ranging from 10 to 500, the simulated dataset was randomly down-sampled, library size normalization was applied, and zero inflated regression models were fit to logcounts for each gene to find DE between conditions using MAST v1.16 (Finak2015-ys).*

7.	Page 7, line 10: The number of individuals being multiplexed in a 10x experiment seems to be increasing especially in the context of population-scale scRNA-seq data (e.g. through new developments likehttps://www.nature.com/articles/s41592-021-01153-z?proof=t%29). Therefore, it would be relevant to the reader to know how the performance is affected with different numbers of samples (10s instead of 5). Please show how the tool performs/compare multiplexing of 10s of individuals with the 5 shown now.

*The flexible nature of splatPop allows for the user to specify the specifics of the population they want to simulate, including the number of individuals multiplexed together, without impacting the performance of the simulation. In Figure 3, we demonstrate simulating 3 donors per batch to make it easy to visualize the results. However, the population simulated to demonstrate sc-eQTL mapping (in Figure 4c,d) included 100 donors simulated in 10 batches, with 10 donors per batch. We have made it clearer in the text that the user can specify any number of individuals to simulate in one batch.*

*Further, to demonstrate that simulating individuals in batches does not substantially impact the speed of splatPop, we added a simulation with 100 individuals in 10 batches to our speed test shown in Table S2.*

8. Page 11, line 20: why specifically this cutoff? Please show benchmarks with commonly used parameters (such as 'expressed in at least 10% of cells in one condition' in MAST, or using all genes that are expressed and show variance in eQTL mapping).

*In the updated version, we use MAST with a FDR 0.05 cutoff as suggested by the reviewer. The updated figure with these results is shown above in response to main comment #6*

Minor points:

-	Page 2, line 36: Perhaps the wording 'top-tier' is a bit overselling the tool. While it is ranked high in the referenced paper, it is not without its downsides and is definitely not top-tier for keeping 'biological signals'.

*We have toned down this wordage as suggested by the reviewer. However, we would also note that we found issues with the way Cao et al. assessed biological signals. From our understanding of the methods in the pre-print, default splat parameters were used to generate the simulations. By default, splat does not simulate biological signals like differential expression in the data. Rather, the user needs to set the parameters to specify that they want DE effects included. We have addressed this at length in response to reviewer 2 (comment #4).*

-	Figure 1: the parameters 'BatchCells' and 'Batch.size' are missing here. Table S1 is also missing 'BatchCells'. Please show how these parameters fit into the model and add 'BatchCells' parameter to Table S1.

*We agree that this information will help readers understand how (and when) batch effects are incorporated into splatPop and have updated Figure 1 and Table S1 as suggested.*

*Relevant part of Figure 1:*

*Relevant part of Table S1:*

-	Page 4, line 18: write out the abbreviation 'BCV' completely the first time it's mentioned. *Addressed.*

-	Page 7, line 31: spelling error 'versus', not 'verses'

*Addressed.*

-	Page 12, line 59: why is a significance cutoff of FDR of 0.10 used, and not the more common significance 0.05 cutoff?

*In the updated version, we use MAST with a FDR 0.05 cutoff as suggested by the reviewer. The updated figure with these results is shown above in response to main comment #6.*

Reviewer #2 :

In this study, the authors proposed a novel simulation method that generate scRNA-seq data with corresponding eQTL effect. The same simulation framework also allows for the simulation of complex batch effects, cell group effects, and conditional effects between individuals from different cohorts. The manuscript is well-written and the models are presented very clearly with visually pleasing figures and reproducible html. While the concept simulating eQTL effect in the model is innovative, we feel the current version of the manuscript as it stands didn't fully validate the simulation model. The manuscript will benefit from further clarity and justification of the model. Below we comment and suggest a number of steps that will add to the manuscript.

*We thank the reviewer for their time in assessing our manuscript and for their thoughtful comments, addressed below.*

1. There are two key components or application aspects of the paper

(a)	Creating a new simulation model that can simulate population-scale scRNA-seq data with corresponding eQTL effect.

(b)	The additional ability of this model to generate scRNA-seq data with more complex intrinsic

structure.

It is important to validate the two aims of this paper separately.

2.	Following on from point 1 (specially component 1b), its important to compare more widely. A recent simulation framework, Muscat*, also enables the simulation of multi-samples across multi-conditions. While this method has a different framework and does not use eQTL data in its modelling, the authors may want to consider citing and discussing this work.

* Crowell, H. L., Soneson, C., Germain, P. L., Calini, D., Collin, L., Raposo, C., ... & Robinson, M. D. (2020). Muscat detects subpopulation-specific state transitions from multi-sample multi-condition single-cell transcriptomics data. Nature communications, 11(1), 1-12.

*We appreciate this suggestion and have added a discussion of Muscat to our introduction:*

*Further, existing multi-sample single-cell simulation frameworks, such as Muscat (Crowell2020), do not incorporate genetic effects (i.e., eQTL) into their simulations, and therefore are limited in their downstream applications.*

3.	In Fig 2a, the authors showed that the inter-individual relationship pattern between empirical and simulated individuals is not the same, and stated it is because the simulated individuals do not have the same genotype as the reference individual. The authors then mentioned, "However, such relationship patterns could be achieved by providing splatPop with the genotype data for the population of interest." As the novelty of splatPop lies in its ability to take in genotype data and simulate realistic population structure, an example case study of the above statement should be provided. Currently, it seems that in majority of case studies (Fig 2, Fig S2-S5), the inter-individual relationship pattern in the simulated data is different to the empirical.

*We thank the reviewer for bringing up the issue of inter-individual relationships in our simulations. Due to the sampling performed by splatPop (i.e. when gene means for each individual are sampled from a gamma distribution parameterized from empirical data), splatPop simulations maintain the population level properties of empirical data, but not the inter-individual relationships. We have replaced the statement quoted by the reviewer above with a more clear description of this as a limitation of the sampling approach used by splatPop:*

*As the simulated individuals are not the same genotypes as the reference individuals and as the base gene means are sampled randomly for each individual, we do not expect the same inter-individual relationship pattern to be observed between simulated and empirical cells. However, splatPop can be used to replicate more exactly an empirical dataset (see “Replicating empirical data with splatPop” below).*

*However this comment from the reviewer (and comment #1 from reviewer #1) encouraged us to extend the functionality of splatPop to allow users to more closely replicate provided empirical data if desired. Now, users can provide the empirical data directly to the splatPopSimulate function causing it to bypass the gene mean and variance sampling steps, and instead use the provided data directly as the base gene means. An example of how to use this feature has been added to the package vignette and the workflowr scripts provided that support this manuscript. In addition, the following new results section and supporting figure have been added to the manuscript:*

*Replicating empirical data with splatPop: Without the addition of co-regulation effects, the expression correlation between genes is centered around zero (Figure 4a). This effect occurs because the mean expression value for each gene for each individual is sampled randomly from a distribution. This sampling approach is fast, flexible (e.g., allowing for the simulation of any number of genes), and it maintains the population-level characteristics of empirical scRNA-seq data, all useful properties for many simulation studies. However, splatPop can also be used to simulate scRNA-seq data that more closely replicates the gene expression correlations seen in empirical data. As described above, splatPop estimates population-wide parameters from user provided population-scale bulk RNA-seq or aggregated scRNA-seq data. However, if this empirical data is provided directly to the splatPopSimulate function, it will be used as the base gene means (i.e. the "Gene mean per sample" Figure 1, left panel). Used in this way, splatPop will generate simulated scRNA-seq data for the individuals and genes in the empirical data that replicates empirical inter-individual relationships (Figure 4b) and patterns of gene expression across individuals (Figure 4c). We note that while cell-group, conditional, and batch effects can still be applied to simulations generated this way, adding simulated eQTL effects may compound with eQTL effects already present in the empirical gene mean data, inflating their effect size.*

*Methods: We note that if empirical gene mean data is provided directly to the splatPopSimulate function, the gene mean and variance sampling steps will be ignored, and the empirical gene means will be used directly.*

*Figure 4 Coregulation and replicating empirical 10x-Neuro data. (a) Pearson's correlation between mean aggregated expression of randomly paired (gray, n=1000) and co-regulated (blue, n=54) eGenes across 100 individuals. The average correlation for each type designated by the large point. (b, c) Comparison of 10x-Neuro (left) and splatPop simulated (right) data using the replication approach. Eight individuals sequenced in the same batch and 358 genes meeting a minimum expression threshold (> 10 counts across all cells) were included. (b) Pairwise Pearson's correlation between individuals across genes, with expression for each gene for each individual calculated as the mean aggregated log counts across cells. Individuals are ordered by hierarchical clustering of the empirical data. (c) Mean aggregated log counts across cells for each individual for the top 35 most highly expressed genes. Genes and individuals are ordered by hierarchical clustering of the empirical data.*

4. The authors referred to a recent benchmark ms on scRNA-seq simulation methods*. From the paper, while Splatter is one of the top-tier methods in general across 13 criteria under data property estimation, it performed medicorely in certain criteria such as cell correlation, mean vs fraction of zero relationship and library size vs fraction zero relationship (Fig 2.). While this seems to be a challenging criteria for many simulation methods in general was splatPop able to address this issue and improve from Splat on some of these criteria?

* Cao, Y., Yang, P., & Yang, J. Y. H. (2021). A benchmark study of simulation methods for single-cell RNA sequencing data. bioRxiv.

*We appreciate this comment from the reviewer as it gives us the opportunity to address limitations of both splatter and of the Cao et al. benchmarking study. Splat performs well in a number of the diverse performance criteria included by Cao et al. (e.g., scalability, library size). However, as the reviewer notes, it performs poorly in others. For a number of these criteria, the reason why splat performs poorly is that the criteria being tested are not the intended function (or the default function) of splat. Specifically:*

*-Splat does not replicate genes and/or cells from the empirical data, but rather simulates genes and cells by sampling from empirically derived distributions. Thus, splat is not expected to replicate the “cell correlation” and “gene correlation” structure of the empirical data. While this might make splat the wrong model choice for some applications, generally this simulation approach allows for greater flexibility and scalability. As the reviewers were both interested in simulations that more closely resemble empirical data, we have updated splatPop to be able to replicate gene mean and variance properties and eQTL effects directly from empirical data (see response to comment #3, though we note that cells are still simulated stochastically).*

*-	By default, splat simulates a homogeneous population of cells, thus the biological signals that may be present in the empirical data, including differential expression (DE), variable (DV), distributed (DD), proportioned (DP), and bimodally distributed (BD), will not be preserved in the simulated data. However, splat/splatPop are flexible and allow for the simulation of these kinds of biological signals by changing the control parameters. For example, DE signals will be present if the user specifies they want more than one cell group simulated. The ability of splatPop to incorporate genetic effects (i.e., eQTL) into the simulations means that splatPop cell populations can have additional biological signals such as DD, DP, and BD.*

*The remaining area where splat performs poorly is the mean-variance relationship. The original author and active maintainer of splatter, Luke Zappia, is aware of this issue and has plans to address it in the future however we feel this is beyond the scope of this manuscript. We note that a strength of splatPop is that it is built on top of splat, thus any methodological improvements made in the splat model will be automatically incorporated into splatPop.*

*As the code used by Cao et al. has not been made publicly available and the paper is still under review, we have not included our detailed comments on their benchmark in the splatPop manuscript. Instead, we have updated our discussion of the benchmark to state:*

*Splatter is flexible, fast, reproducible, and well maintained and, while all simulation frameworks have limitations, was considered one of the top performing models for simulating single-cell RNA-seq data in a recent independent benchmark (Cao2021). [...] Further, existing multi-sample single-cell simulation frameworks, such as Muscat (Crowell2020), do not incorporate genetic effects (i.e., eQTL) into their simulations, and therefore are limited in their downstream applications.*

5. Relating to the previous comment, the benchmark paper utilised a strategy of splitting an empirical data into training and testing subset, using the training to simulate and the testing to evaluate the simulated data. A similar framework could be useful to evaluate the simulated data generated in this study and to complement the visuals. For example, given that the data used for simulation in this study includes eQTL information, are the authors able to compare the eQTL effects generated in the simulated data with the empirical eQTL effects?

*We agree that the training-testing strategy used by Cao et al was useful for benchmarking different scRNA-seq simulations tools and that such an approach could also be used to benchmark population-scale scRNA-seq simulation methods. However, as we are not aware of any other scRNA-seq simulation frameworks that incorporate eQTL effects, we do not have anything to benchmark our performance against. However, this suggestion by the reviewer encouraged us to better demonstrate how well the DE and eQTL effect sizes estimated in our downstream analyses compared to the simulated effect sizes. To accomplish these, we added the following panels to Figure 4, that show the relationship between the simulated (x-axis) and estimated (y-axis) DE deltas (panel b) and eQTL betas (panel e), with the Pearson’s correlation between the simulated and estimated effects for true positives.*

*As we know which genes were simulated with conditional DE effects, we can calculate performance metrics such as true positive rate (TPR) and false discovery rate (FDR) (Figure 5a) and assess the relationship between the estimated and simulated DE effect sizes. [...] For example, with 80 cells simulated per individual, the correlation between the estimated and simulated DE effect sizes for TP genes was 0.64 (Figure 5b). [...] Focusing on eQTL mapping results with 80 cells simulated per individual, the correlation between estimated and simulated eQTL effect sizes was 0.95 (Figure 5e).*

*Figure 5 [...] (b) The relationship between simulated and MAST estimated DE effect sizes (delta) using 80 cells per individual for all genes colored by the DE analysis result (TP, FN, FP, TN). The Pearson's correlation between simulated and estimated deltas for true positive (TP) genes is shown in blue. [...] (e) The relationship between simulated and estimated eQTL effect sizes (beta) for all genes colored by the eQTL mapping result (TP, FN, FP, TN). The Pearson's correlation between simulated and estimated betas for TP genes is shown in blue.*

6. Following on point 5, the authors provided a case study of how splatPop can utilise genetic effects on expression (eQTL) to define cell groups by genetic and non-genetic DE. It would be good to extend this case study by providing a downstream of application of this simulated data. Ie, what can people do with the data besides performing a typical DE analysis and finding DE genes between two groups? This allow the comparison of another downstream performance between empirical and simulated data.

*In addition to demonstrating how splatPop simulations can be used for treatment/conditional DE analysis, we demonstrate the use of splatPop for (1) benchmarking single-cell eQTL mapping and discuss its uses for (2) optimizing experimental design and (3) power analysis. We also show in our DE analysis that the results from DE can be further inspected to make it clear that in addition to testing for DE between conditional groups splatPop can be used to test for DE between cell-groups, we added the following to this section of the results: “We note that this same approach could be used to test for DE genes between cell groups.”*

7.	When using splatPop to evaluate DE and eQTL methods, the authors set the conditional DE effects on 40% of the genes and eQTL effect to 70% of genes. These numbers seem quite high, is there a particular reason for choosing such high percentages? Would setting it to a lower number, say 10%, impact the DE and eQTL results?

*Regarding the proportion of eGenes: In the most recent GTEx report, cis-eQTL were discovered (FDR 1%) for over 18k protein-coding genes or about 95% of genes tested (Science 369 1318-1330, 2020). This highlights that most genes are likely to have eQTL, but that these eQTL are not discovered with small sample sizes because of their small effects. splatPop assigns eQTL effect sizes to each eGene by sampling from a distribution of eQTL effect sizes from empirical data (by default using effect sizes estimated by GTEx). Thus, while 70% may seem like a large proportion, many of these associations are assigned a very small effect size by splatPop and will not be discovered unless very large populations are simulated. We believe this is true to how eQTL associations occur biologically. To make this clearer, we added the following to our description of these results: “Because splatPop samples eQTL effect sizes from a distribution estimated from empirical eQTL mapping data, many of these associations will have small effects that could only be successfully discovered if a large number of donors are simulated and used for mapping.“*

*Regarding the proportion of DE genes: The splat model for simulating DE effects that is used by splatPop is highly flexible, allowing for the user to control both the proportion of genes with DE effects and the distribution of DE effect sizes. By adjusting these controls, splatPop can simulate conditional populations with: small DE effects on few genes, small DE effects on many genes, large DE effects on many genes, large DE effects on many genes, or some genes with large DE effects and others with small effects, etc. We feel these different scenarios all represent different, but real, biological scenarios that users may be interested in. Here, for demonstration purposes, we simulate a dataset with conditional DE effects on 40% of the genes. Because these effects are sampled from a distribution, some are large DE effects and others are small and unlikely to be picked up by statistical testing. Simulating a population with fewer DE genes would mean, for the sake of our demonstration, that we would have to simulate more genes in order to have enough DE genes to show stable performance metrics in Figure 4. While splatPop scales nicely and so this is possible, for the sake of our demonstration, we simulated fewer genes with more DE effects.*

8.	Given that a scRNA-seq typically contains more than 20,000 genes, and that a user may want to simulate genes from all chromosomes rather than from just one chromosome, the authors may want to expand their scalability analysis in Table S2 to include more genes. At the moment, only up to 1000 genes is examined.

*We appreciate the suggestion and have expanded Table S2 to include simulations up to 20k genes.*

9.	(minor) In Table S2, the table legend indicates the time is in minutes. However, from reading the manuscript it should be in seconds?

*Thanks, we have fixed Table S2 and the text to consistently refer to time in minutes.*

10.	The benefit of using eQTL data as an extra source of information to simulate multiple individuals needs to be emphasised in greater detail. May be further discussion and additional case studies.

*We agree that the simulation of genetic effects is a major advantage of splatPop and have emphasized this more in the abstract (“[...] but current scRNA-seq simulation frameworks do not simulate population-scale data with genetic effects”), in the introduction (“Further, existing multi-sample single-cell simulation frameworks, such as Muscat, do not incorporate genetic effects (i.e., eQTL) into their simulations, and therefore are limited in their downstream applications.“), and in the discussion (“The simulation of genetic effects also means splatPop can be used to simulate eQTL mapping populations, greatly expanding the usefulness of this model.”).*

**Second round of review**

**Reviewer 1**

The authors have adequately answered my comments, except for a misunderstanding about comment #4 reviewer 1. The authors have implemented a method to assign genes in close proximity the same eSNP and direction of effect, but what I was referring to in this comment, is a differential co-expression relationship driven by genetics. The easiest implementation would assume that the co-expression strength of the two genes is driven by an eQTL on one of the genes. In a biological context, this could for example describe a gene duo composing of a transcription factor (TF) and its downstream target gene (cis-eGene). The strength by which the TF would then bind to the TFBS of that target gene would be depending on the SNP. What the author has implemented however, could be used to model trans-eQTL effects (i.e. eQTL effects further away from the gene), where for example the genes have a strong co-expression relation ship, causing an eQTL on the cis gene, to also influence a downstream trans gene. Modelling these trans effects would require the the ‘eqtl.cored’ parameter to also go beyond the window (and chromosomes) specified as a parameter. If easily implementable, the above implementation would further broaden the applicability of the tool, but otherwise I am happy with the major improvements of the manuscript.

**Reviewer 2**

I am very pleased with this updated version, and the authors have addressed all my comments. Just a few minor points that will improve the clarity of the manuscript.

1. Figure 2D could have a comparable y-axis across the three sub-panels. It will provide a stronger justification for " mean variance relationship stabilises..." (Page 6 Line 19).

2. The position of results in Figure 2 (is somewhat confusing), where the better and more realistic simulation is happening later. I wonder whether (this probably is an editorial decision) one should reduce the length of simple simulation and possibly move some results into Supplementary. Alternative the statement Page 6 (line 6) should be moving forward to the start of the results section Page 4 line 30 to set the correct expectation.

3. It is nice to see the new results in Figure 4. I found the comparison of empirical and simulated hard to judge via side by side imaging plot. To assess at the individual level, a vectorise (scatter plot) between simulated and empirical correlation will better determine the off-diagonal values. To evaluate at the population level, comparing distribution between the two sets of correlation (empirical vs simulated) is important. Similar comment also applied to Figure 4c (left vs right).

4. In Figure 4b-c, the caption did have clear writing, "Comparison of 10x-Neuro (left) and splatPop simulated (right) data using the replication approach.", but for ease reading, it might be important to place some "small headings" in the figures to indicate which one represent simulated and which one represent empirical.

5. I note Figure 3a is in PCA as opposed to tSNE. When I tested the old version of splatPop to simulate "conditional effect", the resulting tSNE looked quite "circular". It will be nice to add to supplement empirical and simulated tSNE to demonstrate that the simulated tSNE looks similar to what we typically expect in the data. The choice of tSNE is because, in the single-cell field, tSNE or UMAP is not becoming a visualisation standard instead of PCA.

**Authors Response**

**Point-by-point responses to the reviewers’ comments:**

Reviewer #1:

The authors have adequately answered my comments, except for a misunderstanding about comment #4 reviewer 1. The author have implemented a method to assign genes in close proximity the same eSNP and direction of effect, but what I was referring to in this comment, is a differential co-expression relationship driven by genetics. The easiest implementation would assume that the co-expression strength of the two genes is driven by an eQTL on one of the genes. In a biological context, this could for example describe a gene duo composing of a transcription factor (TF) and its downstream target gene (cis-eGene). The strength by which the TF would then bind to the TFBS of that target gene would be depending on the SNP. What the author has implemented however, could be used to model trans-eQTL effects (i.e. eQTL effects further away from the gene), where for example the genes have a strong co-expression relation ship, causing an eQTL on the cis gene, to also influence a downstream trans gene. Modelling these trans effects would require the the ‘eqtl.cored’ parameter to also go beyond the window (and chromosomes) specified as a parameter. If easily implementable, the above implementation would further broaden the applicability of the tool, but otherwise I am happy with the major improvements of the manuscript.

*We appreciate this clarification. In our last round of revisions, we implemented a function to allow for the simulation of genetically driven co-expression between genes as if the genes are both targets of the same TF. The reviewer was requesting a feature where the genetically driven co-expression is driven as if one gene is the TF and the second gene is its target. Because of how differential expression between genes is simulated in splatPop, the implementation of this feature is non-trivial and would require extensive re-coding. As we are actively maintaining and improving splatter and splatPop, we will look into including this as an automated feature in the future, but we believe this is beyond the scope of this manuscript. We do note that because splatPop can simulate single-cell RNAseq data from user provided gene mean data, a user could design their own gene means matrix with these types of TF-target relationships and provide it as input to splatPop.*

Reviewer #2:

I am very please with this updated version, and the authors have addressed all my comments. Just a few minor points that will improve the clarity of the manuscript.

1. Figure 2D could have a comparable y-axis across the three sub-panels. It will provide a stronger justification for " mean variance relationship stabilises..." (Page 6 Line 19).

*Thanks for the helpful suggestion. Panel D in all the relevant figures (Figure 2, S2, S3, S5, S6) has been updated to have a consistent y-axis range.*

2. The position of results in Figure 2 (is somewhat confusing), where the better and more realistic simulation is happening later. I wonder whether (this probably is an editorial decision) one should reduce the length of simple simulation and possibly move some results into Supplementary. Alternative the statement Page 6 (line 6) should be moving forward to the start of the results section Page 4 line 30 to set the correct expectation.

*We are happy to make this change if the editor agrees that the current organization or relative length of the result sections is confusing or suboptimal.*

3. It is nice to see the new results in Figure 4. I found the comparison of empirical and simulated hard to judge via side by side imaging plot. To assess at the individual level, a vectorise (scatter plot) between simulated and empirical correlation will better determine the off-diagonal values. To evaluate at the population level, comparing distribution between the two sets of correlation (empirical vs simulated) is important. Similar comment also applied to Figure 4c (left vs right).

*We have generated the figures requested. In the main figure, we show the pairwise correlation between individuals as a heatmap (Figure 4b) and the log counts per gene with the new scatter plot (Figure 4c). The opposite plots are included as a supplemental figure, with the pairwise correlation between individuals as a scatter plot (Figure S7a) and the log counts per gene for the top expressed genes as a heatmap (Figure S7b).*

4. In Figure 4b-c, the caption did have clear writing, "Comparison of 10x-Neuro (left) and splatPop simulated (right) data using the replication approach.", but for ease reading, it might be important to place some "small headings" in the figures to indicate which one represent simulated and which one represent empirical.

*We have added explicit small headings to the figure to make its interpretation clearer.*

5. I note Figure 3a is in PCA as opposed to tSNE. When I tested the old version of splatPop to simulate "conditional effect", the resulting tSNE looked quite "circular". It will be nice to add to supplement empirical and simulated tSNE to demonstrate that the simulated tSNE looks similar to what we typically expect in the data. The choice of tSNE is because, in the single-cell field, tSNE or UMAP is not becoming a visualisation standard instead of PCA.

*We agree with the reviewer that tSNE and UMAP are more common dimension reduction/visualization approaches for single cell data. In the revised manuscript we have added a new supplemental figure (Figure S8) that shows tSNE plots for all the simulations highlighted elsewhere in the manuscript. Because PCA plots show the global relationships as well as local relationships between cells (which we think is useful for demonstrating the functionality of splatPop) we have kept the PCA plots in the comparison figures.*

*Regarding the reviewer’s comment on the “circular” tSNE plot when simulating the conditional effects using the 10x-IPF data as a reference: This circular phenomenon was occurring because we had simulated fewer cells per individual (nCells=60) than what was present in the empirical data (average nCells=302). While not impacting other comparison analyses, the difference in the number of cells had a major impact on the tSNE plots because, especially with sparse 10x data, tSNE needs a sufficient number of cells in order to find structure in the data. In the revised manuscript, we have updated the conditional effect simulations to have 302 cells per individual and updated Figure S6 accordingly. Provided with this number of cells, the tSNE plots for the 10x-IPF conditional effect simulations look as expected (see Figure S8e).*
